# Prevalence of non-alcoholic fatty liver disease in the Russian Ural Eye and Medical Study and the Ural Very Old Study

**DOI:** 10.1038/s41598-022-12004-y

**Published:** 2022-05-12

**Authors:** Mukharram M. Bikbov, Timur R. Gilmanshin, Rinat M. Zainullin, Gyulli M. Kazakbaeva, Ellina M. Iakupova, Albina A. Fakhretdinova, Azaliia M. Tuliakova, Songhomitra Panda-Jonas, Leisan I. Gilemzianova, Dinar A. Khakimov, Liana A. Miniazeva, Jost B. Jonas

**Affiliations:** 1 Ufa Eye Research Institute, 90 Pushkin Street, Ufa, Russia 450077; 2 Ufa Eye Institute, Ufa, Russia; 3 Privatpraxis Prof Jonas und Dr Panda-Jonas, Heidelberg, Germany; 4 Department of Ophthalmology, Medical Faculty Mannheim, Heidelberg University, Theodor-Kutzerufer 1, 68167 Mannheim, Germany; 5 Institute of Molecular and Clinical Ophthalmology Basel, Basel, Switzerland

**Keywords:** Non-alcoholic fatty liver disease, Public health

## Abstract

Information about prevalence and associated factors of non-alcoholic fatty liver disease (NAFLD) has been scarce for the Russian, Eastern European and Central Asian world region. We assessed prevalence and associated factors of NAFLD in two population-based studies (Ural Eye and Medical Study (UEMS), Ural Very Old Study (UVOS)), which were conducted in rural and urban regions in Bashkortostan/Russia and included participants aged 40 + years and 85 + years, respectively. Defining NAFLD by an absence of regular alcohol consumption, and by abnormally high alanine transaminase (ALT) and aspartate transaminase (AST) concentrations or an AST/ALT ratio of > 1.0, 2341 out of 5852 UEMS participants (40.0%; 95% confidence intervals (CI) 38.8, 41.3) had NAFLD. A higher NAFLD prevalence correlated (multivariable analysis) with older age (odds ratio (OR) 1.02; 95%CI 1.01, 1.03; *P* < 0.001), female sex (OR 1.87; 95%CI 1.58, 2.21; *P* < 0.001), higher waist-hip circumference ratio (OR 2.64; 95%CI 1.11, 6.27; *P* = 0.03), lower depression score (OR 0.98; 95%CI 0.96, 0.999; *P* = 0.04), higher serum concentrations of creatinine (OR 1.004; 95%CI 1.000, 1.008; *P* = 0.03) and bilirubine (OR 1.009; 95%CI 1.002, 1.015; *P* = 0.008), lower prothrombin index (OR 0.99; 95%CI 0.985, 0.998; *P* = 0.01), lower ankle-brachial index (OR 0.49; 95%CI 0.32, 0.75; *P* = 0.001), higher prevalence of a grain-rich diet (OR 1.88; 95%CI 1.50, 2.36; *P* < 0.001) and iron deficiency-related anemia (OR 1.61; 95%CI 1.13, 2.29; *P* = 0.009), and lower prevalence of vigorous leisure activities (OR 0.84; 95%CI 0.72, 0.99; *P* = 0.04). In the UVOS, NAFLD prevalence (mean: 789/1130; 69.8%; 95%CI 67.1, 72.3) was associated with female sex (OR 2.24; 95%CI 1.66, 3.01; *P* < 0.001), higher serum concentrations of low-density lipoproteins (OR 1.34; 95%CI 1.17, 1.55; *P* < 0.001), lower prothrombin index (OR 0.98; 95%CI 0.96, 0.99; *P* = 0.002), and lower ankle-brachial index (OR 0.03; 95%CI 0.02, 0.29; *P* = 0.003). The NAFLD prevalence of 40% in the UEMS and 69.8% in the UVOS corresponds to findings obtained in other world regions and shows the importance of NAFLD, including its determinants such as age, sex, waist-hip ratio, serum creatinine concentration, prothrombin index, ankle-brachial index, and lower physical activity.

## Introduction

Non-alcoholic fatty liver disease (NAFLD) is characterized by a storage of fat in the liver without a clear cause such as alcohol consumption. NAFLD has also been termed metabolic dysfunction-associated fatty liver disease (MAFLD)^1,2^. NAFLD and alcoholic liver disease are types of the overall fatty liver disease spectrum. Within the group of NAFLD, the type of non-alcoholic steatohepatitis (NASH), as compared to the less dangerous non-alcoholic fatty liver, shows signs of liver inflammation, and may progress to liver cirrhosis, liver cancer and liver failure. It may lead to other systemic complications including cardiovascular disease, arterial hypertension, and chronic kidney disease^1–10^. The prevalence of NAFLD as the worldwide most common liver disorder has been estimated to be 25% in the global population^2–6,11^. It is found relatively frequently in countries with a high socioeconomic index, such as the United States, where it affected approximately 75 to 100 million individuals in the year 2017^3–7^. In a recent study conducted by Ciardullo and colleagues in the United States, the prevalence of NAFLD and MAFLD in the 2017–2018 National Health and Nutrition Examination Survey was 37.1% and 39.1%, respectively, with a higher prevalence among Hispanic individuals^11^. The NAFLD prevalence is even more common in countries of the Middle East region and in Turkey^5,12–14^. Major risk factors for NAFLD are obesity and type 2 diabetes, besides body overweight, metabolic syndrome, a diet high in fructose, and older age^1,3–8^. The diagnosis of NAFLD, NASH and the more progressed stages of liver diseases including cirrhosis profoundly depend on the histological results of liver biopsies in the clinical examinations, while in routinely performed medical examinations, the definition of NAFLD is usually based on the measurements of the so called liver enzymes, such as alanine aminotransferase (ALT) and aspartate aminotransferase (AST), and on a synopsis of NAFLD-related risk factors like body mass index, sex, age, blood platelet count and others^1,3,4,8^. It has led to the formulation of risk indices such as the AST/ALT ratio, the BARD index, the pediatric NAFLD fibrosis index, and the liver fibrosis index^15–17^.

Most of the information about the prevalence of NAFLD and related disorders and their associations with other diseases such chronic kidney disease and cardiovascular disease come from study samples which were not recruited on the basis of a general population. In addition, there is almost no information available about the prevalence of NAFLD and its associations in Russia although Russia is by its geographic size the largest, and by population one of the biggest countries globally. We therefore conducted the present population-based studies to assess the prevalence of NAFLD and related disorders in rural and urban populations in Bashkortostan, the most populous republic in Russia. We additionally determined the presence of other major diseases in the study populations to explore associations between NAFLD and other diseases in a population-based manner.

## Methods

The individuals included into the present study were the participants of the Ural Eye and Medical Study (UEMS) and the Ural Very Old Study (UVOS). The UEMS is a population-based investigation which was performed in the Russian republic of Bashkortostan at the southwestern end of the Ural Mountains in the study period from 2015 to 2017^18,19^. Study regions were Ufa as capital of Bashkortostan in a distance of about 1400 km East of Moscow and a rural region in the Karmaskalinsky District in a distance of 65 km from Ufa. The republic of Bashkortostan located between the Volga River and the Ural Mountains, is the most populous republic in Russia with a population of 4 million people. Inclusion criteria for the study were living in the study regions and an age of 40 years or older. The Ethics Committee of the Academic Council of the Ufa Eye Research Institute approved the study design and confirmed that the study adhered to the Declaration of Helsinki, and all participants gave an informed written consent. As described in detail recently, out of a total group of 7328 eligible individuals, 5899 (80.5%) individuals (3319 [56.3%] women) with a mean age of 59.0 ± 10.7 years (range: 40–94 years) participated in the study^18,19^. The study population did not differ significantly in the gender and age distribution from the Russian population as explored in the census carried out in 2010^20^.

The UVOS is a population-based study, which was conducted in the period from 2017 to 2020 in similar, but not the same, study regions as the UEMS was performed^21^. The study was approved by the Ethics Committee of the Academic Council of the Ufa Eye Research Institute and informed written consent was obtained from all participants. Inclusion criteria were an age of 85 + years and living in the study regions. Out of 1882 eligible inhabitants aged 85 + years and living in the study regions, 1526 (81.1%) persons participated in the study. The eligible individuals included the inhabitants of three private small retirement homes in the urban study region. There were no retirement homes in the rural study region. As already described recently, the participation rate did not vary markedly between the urban group (1238 (81.3%) out of 1523 individuals) and the rural group (288 (80.2%) out of 359 individuals)^21^. According to the census carried out in Russia in 2010, the composition of the population of the UVOS with respect to gender and age corresponded to the gender and age distribution in the Russian population beyond an age of 85 + years, with a marked preponderance of females^20^.

Using a bus, the study participants in both studies were brought from their homes to the Ufa Eye Institute where a team of about 20 trained medical doctors and technicians performed all examinations. Those UVOS participants being too immobile for the transport, were examined at their homes. As also described in detail previously, the series of examinations started with a detailed interview consisting of more than 250 standardized questions on the socioeconomic background, including the self-reported ethnicity, level of education, occupation, family income and family estate (ownership of a house and second house, telephone, smartphone, laptop, television, bicycle and car), and size and structure of the family; diet (number of meals per day, frequency and amount of intake of vegetables, fruits, whole grain and meat, consumption of tea and coffee, use of animal fat or cooking oil); smoking (since when or stopped, cigarettes or other types of tobacco products, symptoms of smoking cessation); alcohol consumption (since when or stopped, alcohol consumption-related wrongdoing); physical activity (frequency and intensity of daily work, leisure time activities, sitting or reclining); quality of life and quality of vision; symptoms of chronic obstructive pulmonary disease (COPD), asthma, kidney disease and orthopedic disorders; history of any type of injuries and inter-personal violence; and health assessment questions^18,21^. The questionnaire additionally included questions on the medical history including known diagnosis and therapy of major disorders such as diabetes mellitus, arterial hypertension, cardiovascular diseases, headache, neck pain, thoracic spine and low back pain, depression, suicidal ideas, anxiety, questions on previous neurologic attacks including stroke, epilepsy, polyneuropathy and unconsciousness, and questions on cognitive function and hearing loss. The questions included in the interview were taken from standardized interviews published in the literature, such as the “Center for Epidemiologic Studies Depression Scale (CES-D) Scoresheet”, the Folstein test, Zung´s self-rated depression scale, the National Eye Institute Visual Functioning Questionnaire-25 (VFQ-25), the Questionnaire for Verifying Stroke-Free Status (QVSFS) from the American Heart Association, and the Michigan Neuropathy Screening Instrument^18,21^.

The examinations further included anthropometry, blood pressure measurement, handgrip dynamometry, spirometry, and biochemical analysis of blood samples taken under fasting conditions. We defined arterial hypertension according to the criteria published by the American Heart Association, and criteria for the diagnosis of diabetes mellitus were a fasting glucose concentration of ≥ 7.0 mmol/L or a self-reported history of physician diagnosis of diabetes mellitus or a history of drug treatment for diabetes (insulin or oral hypoglycemic agents). Anemia was defined by a hemoglobin concentration of < 140 g/L for men and < 130 g/L for women. Depression was assessed by applying the Center for Epidemiologic Studies Depression Scale (CES-D) Scoresheet^22^. The estimated glomerular filtration rate (eGFR) was calculated using the chronic kidney disease (CKD) Epidemiology Collaboration (CKD-EPI) equation^23^. The Guidelines for Accurate and Transparent Health Estimates Reporting (GATHER statement guidelines) for collecting the data were applied^24^ We defined NAFLD by abnormally high ALT and AST concentrations or by an AST/ALT ratio of > 1.0, in the absence of alcohol consumption on a regular basis, and in absence of a long-term intake of steatogenic medication such as tetracyclines, valproic acid, amiodarone, methotrexate, highly active antiretroviral therapy, and tamoxifen.

Inclusion criteria for the present study were the availability of measurements of AST and ALT. Using a statistical software package (SPSS for Windows, version 27.0, IBM-SPSS, Chicago, IL, USA), we assessed the main outcome parameters, i.e., the prevalence of NAPLD (expressed as mean and 95% confidence intervals (CI)), and searched for associations between the NAFLD prevalence and other parameters, first in univariable binary regression analyses and followed by multivariable binary regression analyses. The latter included the NAFLD prevalence as dependent variable and as independent parameters all those variables which were associated with the NAFLD prevalence in the univariable analysis with a *P* value of ≤ 0.10. In a step-by-step manner, we dropped those parameters out of the list of independent parameters when they were no longer significantly associated with the NAFLD prevalence. We then added parameters, which had previously dropped out, again to the model to test for the significance of their potential association with the NAFLD prevalence. We calculated the odds ratio (OR) and the 95% CIs. All *P* values were two-sided and were considered statistically significant when the values were less than 0.05.

## Results

Out of 5899 participants of the UEMS, the present investigation included 5852 (99.2%) individuals (3298 [56.4%] women) with measurements of the ALT and AST concentrations. The mean age was 59.0 ± 10.7 years (median: 58 years; range: 40–94 years). The group of study participants as compared with the group of individuals without measurement of the liver enzymes did not differ significantly in age (*P* = 0.26) and sex (*P* = 0.14).

The mean ALT concentration was 21.2 ± 12.1 IU/L (median: 19.4 IU/L; range: 0.9–371 IU/L), and the mean AST concentration was 20.8 ± 11.0 IU/L (median: 19.4; range: 0.7–413 IU/L). An ALT concentration higher than 40 IU/L in men or > 31 IU/L in women was measured for 469/5852 (8.0%), and an AST concentration of > 37 IU/L in men and > 31 IU/L in women was found in 463/5852 (7.9%) individuals. Both, the ALT concentration (21.9 ± 12.1 IU/L versus 20.7 ± 12.0 IU/L; *P* = 0.001) and the AST concentration (21.3 ± 10.2 IUI/L versus 20.4 ± 11.6 IU/L; *P* < 0.001) were significantly higher in men than in women. The mean AST/ALT ratio was 1.04 ± 0.49 (median: 0.96; rage: 0.06–19.1). An AST/ALT ratio of > 1.0 was found in 2623/5852 (44.8%) participants. Out of the whole study population, 1241 participants indicated to consume alcohol regularly.

**Figure 1:**
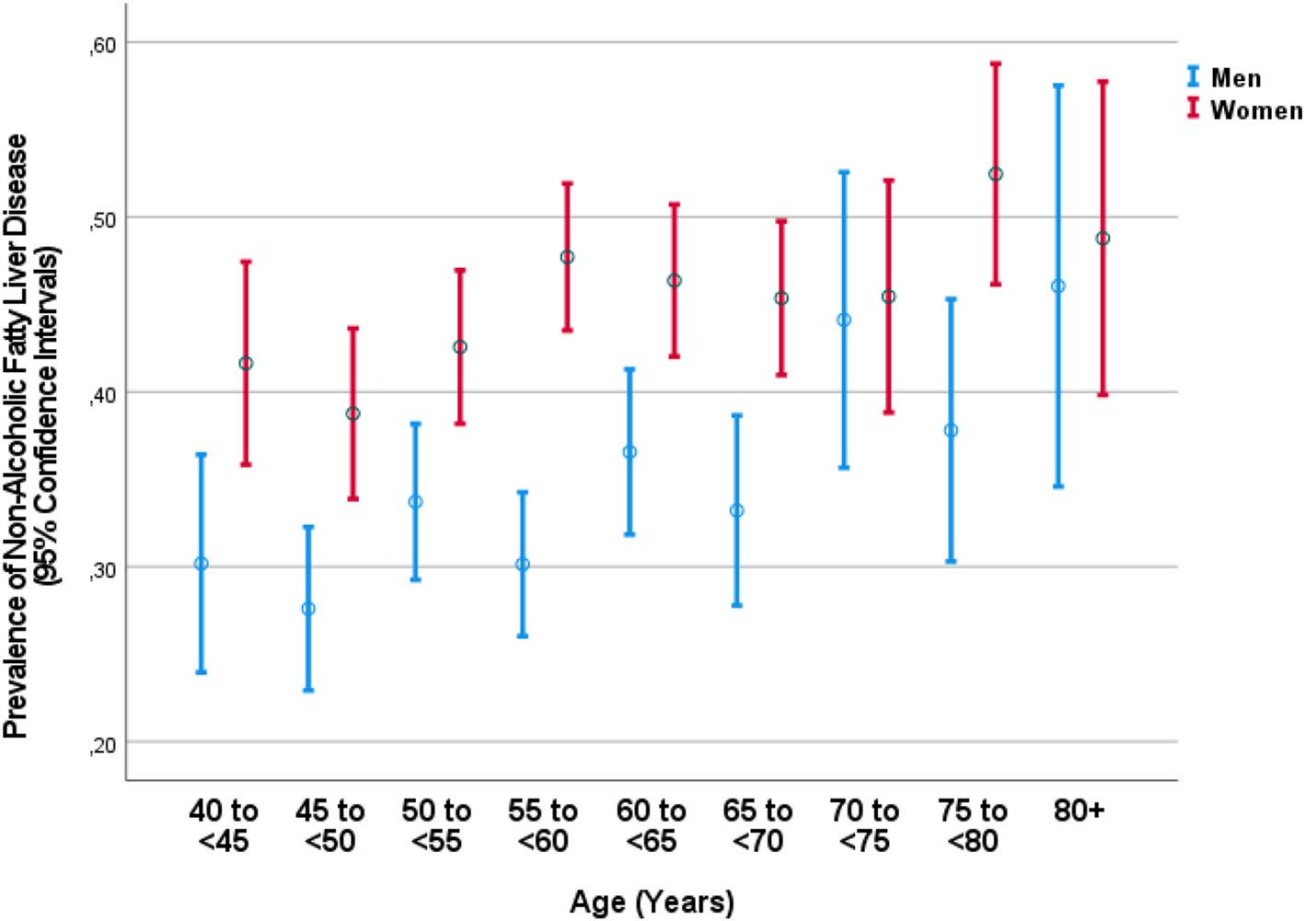
Graph showing the distribution of the prevalence of non-alcoholic fatty liver disease in the Ural eye and Medical Study, stratified by age and sex.

The prevalence of NAFLD was 2341/5852 or 40.0% (95%CI 38.8, 41.3). In univariable analysis, a higher NAFLD prevalence was associated (*P* ≤ 0.10) with older age, higher female sex (Fig. 1), non-Russian ethnicity, lower body height and body weight, lower prevalence of current smoking and lower smoking package years, higher intake of food containing grain, lower reported salt consumption, lower degree of meat processing, lower prevalence of vigorous or moderate activity at leisure times, shorter time spent sitting or reclining, higher prevalence of a positive history of arterial hypertension, arthritis, cardiovascular diseases including stroke, iron-deficiency anemia, osteoarthritis, thyroid disease, unconsciousness and menopause, higher concentration of bilirubine and triglycerides, lower concentration of hemoglobin, a faster erythrocyte sedimentation rate, a lower estimated glomerular filtration rate, longer blood clotting time, lower erythrocyte count, lower percentage of segment nuclear granulocyte and lymphocytes, higher prevalence of anemia, lower diastolic blood pressure, lower ankle-brachial index, higher prevalence of the metabolic syndrome, and lower dynamometric hand grip force (Table 1).

**Table 1:** Associations (univariate analysis) between the prevalence of non-alcoholic fatty liver disease (NAFLD) and other parameters in the Ural Eye and Medical Study.

	NAFLD	No NAFLD	*P* value
Age (years)	59.9 ± 10.8	58.3 ± 10.5	< 0.001
Gender (men/women)	855/1486	1699/1812	< 0.001
Region of habitation (rural/urban)	1343/998	2044/1467	0.52
Ethnicity (non-Russian/Russian)	1672/511	2512/671	0.045
Body height (cm)	163.8 ± 8.6	165.5 ± 8.8	< 0.001
Body weight (kg)	75.3 ± 14.5	76.3 ± 14.6	0.008
Body mass index (kg/m^2^)	28.1 ± 5.0	27.9 ± 5.0	0.12
Waist circumference (cm)	94.1 ± 13.1	94.0 ± 13.5	0.79
Hip circumference (cm)	103.7 ± 12.4	103.6 ± 12.7	0.62
Waist/hip circumference ratio	0.91 ± 0.09	0.91 ± 0.09	0.94
Level of education	5.60 ± 1.48	5.63 ± 1.42	0.38
Socioeconomic score	5.84 ± 1.57	5.89 ± 1.50	0.20
Smoking, currently	197/2341 (8.4%)	538/3511 (15.3%)	< 0.001
Smoking package years	2.5 ± 10.0	5.1 ± 14.1	< 0.001
Alcohol consumption, any	0	1241/3511 (35.4%)	< 0.001
Number of daily meals	3.64 ± 0.80	3.62 ± 0.79	0.42
In a week how many days do you eat fruits?	5.37 ± 2.00	5.35 ± 1.97	0.72
In a week how many days do you eat vegetables?	6.23 ± 1.44	6.28 ± 1.40	0.12
Type of oil for cooking used: vegetable cooking oil–animal fat (butter)	1752/51	2685/99	0.20
Food containing whole grains (yes/no)	1795/387	2474/707	< 0.001
Salt consumed per day (g)	4.19 ± 2.33	4.31 ± 2.38	0.06
Degree of processing meat (weak–medium–strong)	53/734/1392	66/1176/1940	0.09
In your leisure time, do you do any physically vigorous activities like running, strenuous sports or weight lifting for at least 10 min at a time? (yes/no)	600/1682	1056/2394	< 0.001
In your leisure time, do you do any moderate intensity activities like brisk walking, cycling or swimming for at least 10 min at a time?	1023/1228	1682/1737	0.006
Over the past 7 days, how much time did you spend sitting or reclining on a typical day? (minutes)	1004 ± 814	1109 ± 986	< 0.001
History of angina pectoris	212/2341 (9.1%)	320/3511 (9.1%)	0.96
History of asthma	64/2341 (2.7%)	98/3511 (2.8%)	0.94
History of arterial hypertension	997/2341 (42.6%)	1359/3511 (38.7%)	0.003
History of arthritis	697/2341 (30.5%)	920/3511 (26.2%)	0.003
History of previous bone fractures	666/2183 (30.5%)	970/3183 (30.5%)	1.00
History of low back pain	1184/2183 (54.2%)	1713/3183 (53.8%)	0.78
History of thoracic spine pain	534/2183 (24.5%)	726/3183 (22.8%)	0.17
History of neck pain	655/2183 (30.0%)	905/3183 (28.4%)	0.22
History of headache	1043/2183 (47.8%)	1492/3183 (46.9%)	0.52
History of cancer	73/2341 (3.1%)	101/3511 (2.9%)	0.64
History of cardiovascular disorders including stroke	627/2183 (28.7%)	830/3183 (26.1%)	0.03
History of dementia	18/2183 (0.8%)	19/3183 (0.6%)	0.32
History of diabetes mellitus	208/2341 (8.9%)	286/3511 (8.1%)	0.34
History of diarrhea	13/2183 (0.6%)	14/3183 (0.4%)	0.44
History of iron-deficiency anemia	151/2183 (6.9%)	152/3183 (4.8%)	0.001
History of low blood pressure and hospital admittance	80/2332 (3.4%)	134/3492 (3.8%)	0.44
History of osteoarthritis	448/2183 (20.5%)	532/3183 (16.7%)	< 0.001
History of skin disease	128/2183 (5.9%)	154/3183 (4.8%)	0.11
History of thyroid disease	262/2341 (11.2%)	343/3511 (9.8%)	0.09
History of falls	460/2341 (19.7%)	639/3511 (9.8%)	0.17
History of unconsciousness	221/2341 (19.7%)	265/3511 (7.5%)	0.01
History of menopause	1121/1353 (82.9%)	1222/1552 (78.7%)	0.005
Age of the last regular menstrual bleeding (years)	48.2 ± 5.1	48.1 ± 4.9	0.58
Age of last menstrual bleeding (years)	48.4 ± 5.1	48.3 ± 4.9	0.74
Alanine aminotransferase (IU/L)	22.2 ± 15.9	20.6 ± 8.5	< 0.001
Aspartate aminotransferase (IU/L)	23.8 ± 14.2	18.8 ± 7.6	< 0.001
Aspartate aminotransferase/alanine aminotransferase ratio	1.18 ± 0.62	0.94 ± 0.34	< 0.001
Bilirubine, total (µmol/L)	15.5 ± 12.2	14.5 ± 10.5	0.001
High-density lipoproteins (mmol/L)	2.34 ± 0.93	2.30 ± 0.87	0.22
Low-density lipoproteins (mmol/L)	2.12 ± 1.18	2.14 ± 1.21	0.68
Cholesterol (mmol/L)	5.82 ± 1–62	5.77 ± 1.73	0.22
Triglycerides (mmol/L)	1.43 ± 0.82	1.40 ± 0.70	0.10
Rheumatoid factor (IU/mL)	0.12 ± 0.94	0.11 ± 0.94	0.58
Erythrocyte sedimentation rate (mm/hour)	14.6 ± 11.4	13.9 ± 11.2	0.02
Glucose (mmol/L)	5.03 ± 1.68	5.03 ± 1.66	0.93
Creatinine (µmol/L)	89.9 ± 26.3	89.9 ± 24.1	0.98
Estimated glomerular filtration rate (mL/min per 1.73 m^2^)	71.0 ± 19.6	73.2 ± 18.7	< 0.001
Urea (mmol/L)	5.12 ± 1.44	5.10 ± 1.48	0.70
Residual nitrogen (g/L)	0.25 ± 0.05	0.25 ± 0.08	0.98
Total protein (g/L)	75.9 ± 6.2	76.0 ± 6.4	0.34
International normalized ratio (INR)	1.07 ± 0.14	1.06 ± 0.15	0.15
Blood clotting time (minutes)	3.79 ± 0.52	3.73 ± 0.54	< 0.001
Prothrombin time (%)	95.8 ± 10.4	96.2 ± 10.1	0.15
Hemoglobin	141.4 ± 14.5	143.4 ± 15.0	< 0.001
Erythrocytes (10^6^ cells/µL)	4.45 ± 0.37	4.51 ± 0.38	< 0.001
Leukocytes (10^9^ cells/L)	5.12 ± 1.46	5.12 ± 1.41	0.90
Rod-core granulocyte (% of leukocytes)	2.47 ± 1.56	2.50 ± 1.57	0.51
Segment nuclear granulocyte (% of leukocytes)	59.0 ± 7.3	59.5 ± 7.5	0.02
Eosinophil granulocytes (% of leukocytes)	2.2 ± 1.1	2.1 ± 1.3	0.41
Lymphocytes (% of leukocytes)	32.1 ± 6.3	31.7 ± 6.3	0.01
Monocytes (% of leukocytes)	5.2 ± 2.1	5.3 ± 2.4	0.19
Prevalence of diabetes mellitus	274/2341 (11.7%)	407/3511 (11.6%)	0.90
Anemia (serum hemoglobin concentration < 140 g/L in men, < 130 g/L in women)	593/2341 (25.3%)	791/3511 (22.5%)	0.01
Blood pressure, systolic	133.8 ± 20.9	133.4 ± 20.2	0.41
Blood pressure, diastolic	81.7 ± 10.4	82.2 ± 10.4	0.07
Blood pressure, mean	99.1 ± 12.6	99.3 ± 12.5	0.58
Arterial hypertension	2058/2341 (87.9%)	3130/3511 (89.2%)	0.15
Arterial hypertension, stages	2.16 ± 1.05	2.18 ± 1.02	0.62
Prevalence of chronic obstructive pulmonary disease	140/2180 (6.4%)	228/3181 (7.2%)	0.30
Ankle-brachial index, right	1.24 ± 0.19	1.25 ± 0.18	0.01
Ankle-brachial, left	1.23 ± 0.19	1.24 ± 0.18	0.03
Metabolic syndrome	676/2341 (28.9%)	890/3511 (25.4%)	0.003
Hearing loss score	5.18 ± 11.1	5.12 ± 10.9	0.85
Depression Score	1.17 ± 3.73	1.19 ± 3.76	0.85
State-trait anxiety inventory	− 0.60 ± 3.58	− 0.70 ± 3.52	0.32
Manual dynamometry, right hand	28.7 ± 11.3	31.8 ± 11.8	< 0.001
Manual dynamometry, left hand	25.2 ± 11.0	28.1 ± 11.4	< 0.001

In the binary multivariable regression analysis with the NAFLD prevalence as dependent variable, we dropped due to a lack of statistical significance the parameters of prevalence of a history of thyroid disease (*P* = 0.99), ethnicity (*P* = 0.98), body weight (*P* = 0.95), smoking package years (*P* = 0.72), history of arthritis (*P* = 0.67), right ankle-brachial index (*P* = 0.51), blood clotting time (*P* = 0.89), erythrocyte count (*P* = 0.56), prevalence of metabolic syndrome (*P* = 0.48), hemoglobin concentration (*P* = 0.65), history of cardiovascular disease (*P* = 0.58), body height (*P* = 0.44), prevalence of moderate physical activity in leisure time (*P* = 0.46), prevalence of osteoarthritis (*P* = 0.34), erythrocyte sedimentation rete (*P* = 0.32), serum concentration of triglycerides (*P* = 0.41), self-reported salt consumption (*P* = 0.27), degree of meat processing (*P* = 0.12), lymphocyte count (*P* = 0.23), segment nuclear leukocyte count (*P* = 0.71), history of arterial hypertension (*P* = 0.10), prevalence of anemia (*P* = 0.26), estimated glomerular filtration rate (*P* = 0.14), history of unconsciousness (*P* = 0.06), bilirubine concentration (*P* = 0.06), and dynamometric hand grip force (in exchange for sex). When we added the parameters of serum concentrations of high-density lipoproteins (*P* = 0.70), low-density lipoproteins (*P* = 0.39), triglycerides (*P* = 0.48), cholesterol (*P* = 0.86), glucose (*P* = 0.49) and hemoglobin (*P* = 0.91), total protein content (*P* = 0.28), and body mass index (*P* = 0.56) as single parameters to the model, they were not significantly correlated with the NAFLD prevalence. In contrast, the parameters of serum concentration of creatinine and bilirubine, the prothrombin index, waist-hip circumference ratio, depression score, arterial hypertension stage and ankle-brachial index were, when added to the model, significantly associated with the NAFLD prevalence. In the final model, a higher NAFLD prevalence correlated with older age, female sex, higher waist-hip circumference ratio, lower depression score, higher serum concentrations of creatinine and bilirubine, lower prothrombin index, lower ankle-brachial index, lower arterial hypertension stage, higher prevalence of a grain-rich diet and of iron deficiency-related anemia, and lower prevalence of vigorous leisure activities (Table 2). If the prothrombin index was replaced by the INR value, the latter was associated (OR 1.73; 95%CI 1.09, 2.72; *P* = 0.02).

**Table 2:** Associations (multivariable analysis) between the prevalence of non-alcoholic fatty liver disease (NAFLD) and other parameters in the Ural Eye and Medical Study.

	Odds ratio	95% Confidence interval	*P* value
Age (years)	1.02	1.01, 1.03	< 0.001
Sex (men/women)	1.87	1.58, 2.21	< 0.001
Waist-hip circumference ratio	2.64	1.11, 6.27	0.03
Depression score	0.98	0.96, 0.999	0.04
Creatinine serum concentration (mmol/L)	1.004	1.000, 1.008	0.03
Bilirubine serum concentration (mmol/L)	1.009	1.002, 1.015	0.008
Prothrombin index	0.99	0.985, 9.998	0.01
Ankle-brachial index	0.49	0.32, 0.75	0.001
Arterial hypertension stage	0.89	0.82, 0.96	0.003
Food containing grains	1.88	1.50, 2.36	< 0.001
History of iron-deficiency anemia	1.61	1.13, 2.29	0.009
In your leisure time, do you do any physically vigorous activities like running, strenuous sports or weight lifting for at least 10 min at a time?	0.84	0.72, 0.99	0.04

Out of 1526 participants of the Ural Very Old Study, measurements of the ALT and AST were available for 1130 (74.0%) individuals (298 (26.4%) men) with a mean age of 88.2 ± 2.8 years (range: 85–100 years). The group of study participants as compared with the group of individuals without measurement of the liver enzymes was significantly younger (88.2 ± 2.8 years versus 88.6 ± 3.1 years; *P* = 0.03), while both groups did not differ significantly in sex (*P* = 0.22).

The mean ALT concentration was 17.4 ± 8.7 IU/L (median: 16.4 IU/L), and the mean AST concentration was 25.2 ± 10.1 IU/L (median: 24.5 IU/L). An ALT concentration higher than 40 IU/L in men or > 31 IU/L in women was measured for 49/1130 (4.3%), and an AST concentration of > 37 IU/L in men and > 31 IU/L in women was found in 257/1130 (22.7%). Both, the ALT concentration (17.7 ± 8.8 IU/L versus 17.3 ± 8.6 IU/L; *P* = 0.49) and the AST concentration (25.8 ± 9.7 IUI/L versus 25.0 ± 10.2 IU/L; *P* = 0.23) did not differ significantly between men and women. The mean AST/ALT ratio was 1.84 ± 1.51 (median: 1.42; rage: 0.16–21.61). An AST/ART ratio of > 1.0 was found in 859/1130 (76.0%) participants. Defining NAFLD by an absence of consuming alcohol on a regular basis, and by abnormally high ALT and AST concentrations or by an AST/ALT ratio of > 1.0, 789/1130 (69.8%; 95%CI 67.1, 72.3) individuals had a positive result. In multivariable analysis, a higher NAFLD prevalence was associated with female sex (OR 2.24; 95%CI 1.66, 3.01; *P* < 0.001), higher serum concentrations of low-density lipoproteins (OR 1.34; 95%CI 1.17, 1.55; *P* < 0.001), lower prothrombin index (OR 0.98; 95%CI 0.96, 0.99; *P* = 0.002), and lower ankle-brachial index (OR 0.03; 95%CI 0.02, 0.29; *P* = 0.003) (Table 3).

**Table 3:** Associations (multivariable analysis) between the prevalence of non-alcoholic fatty liver disease (NAFLD) and other parameters in the Ural Very Old Study.

	Odds ratio	95% Confidence interval	*P* value
Sex (men/women)	2.24	1.66, 3.01	< 0.001
Low-density lipoprotein serum concentration (mmol/L)	1.34	1.17, 1.55	< 0.001
Prothrombin index	0.98	0.96, 0.99	0.002
Ankle-brachial index	0.03	0.02, 0.29	0.003

## Discussion

In our population-based investigations from Russia, the prevalence of NAFLD was 2341/5852 (40.0%) in the UEMS and 789/1130 (69.8%) in the UVOS. In the UEMS, determinants of a higher NAFLD prevalence were factors such as older age, female sex, higher waist-hip circumference ratio, lower depression score, higher serum concentrations of creatinine and bilirubine, lower prothrombin index, lower ankle-brachial index, and lower prevalence of vigorous leisure activities. In the UVOS, a higher NAFLD prevalence was associated with female sex, higher serum concentrations of low-density lipoproteins, a lower prothrombin index, and lower ankle-brachial index.

The findings made in both of our studies agree with observations made in previous studies in other populations. Single studies and meta-analyses estimated the global NAFLD prevalence to be 25%^2–6,11,12,25–27^. This figure is roughly comparable to the NAFLD prevalence of 40% found in our study population, if one takes into account that in our study the minimal age was 40 years and that the NAFLD prevalence increased with older age (Fig. 1). Subsequently, the NAFLD prevalence was higher in the UVOS study than in the UEMS study. In addition, the NAFLD prevalence depends on the world region, with the reported highest prevalence in the Middle East and South America and lowest in Africa^5,12^. The most comprehensive, so far available study from Russia on the NAFLD prevalence was the DIREG study on individuals treated in outpatient departments of Russian hospitals. In that study population, the mean NAFLD prevalence was approximately 37% to 42% with a maximum of 56.9% in Novosibirsk and a rate of 40% in Bashkortostan, the republic where the current studies were carried out^28,29^. One has to take into account that the DIREG study was not population-based and that the mean age of the study ranged from 12 to 85 years, while in our study the minimum age was 40 years. In the preceding DIREG-1 study on patients aged 18 to 80 years and attending various polyclinics for ambulatory therapeutic care (with or without apparent signs of hepatic diseases) at 208 centers in Russia in the year 2007, the prevalence of NAFLD as detected by sonography was 27.0%^30^.

As in previous studies, a higher NAFLD prevalence in our study population was associated with parameters such as older age and female sex (Fig. 1)^3–10^. The association with older age was not significant in the UVOS, probably due to the high age and the relatively small age range of the study population. In the study populations of both the UEMS and the UVOS as well as in previous study cohorts, women as compared to men had a higher NAFLD prevalence^3–9^. In the UEMS, a higher NAFLD prevalence was additionally correlated with a higher waist-hip circumference ratio, fitting with previous findings of associations between obesity and NAFLD. In that context one may consider that NAFLD can also occur in the absence of obesity, as it was initially described for Asian populations. It may explain why the odds ratio for the association between the waist-hip circumference ratio and the NAFLD was not markedly high in our study population (OR 2.64; 95%CI 1.11, 6.27; *P* = 0.03) (Table 2). The correlation between a higher NAFLD prevalence and a higher serum concentration of creatinine corresponds to the reported complications of NAFLD including chronic kidney disease^9^. Interestingly, the NAFLD prevalence was not correlated with the estimated glomerular filtration rate in our study populations, fitting with the observations made in the Hispanic Community Health Study/Study of Latinos in which elevated serum aminotransferase levels were not associated with a low eGFR in a multivariable analysis^31^. The correlation between the NAFLD prevalence and a lower prothrombin index may suggest a reduced liver function in some of our study participants with NAFLD. The association between a higher NAFLD prevalence and a lower ankle-brachial index as found in both of our study populations may reflect the increased risk of vascular complications in patients with NAFLD and related risk factors. Finally, the association between a higher NFAFLD prevalence and a lower prevalence of vigorous leisure activities reflects the correlation between lower physical activities and the risk factors for NAFLD.

The findings made in our study populations indicate, in agreement with the previous investigations performed in other world regions, the public health importance of NAFLD in Russia. It holds true in particular since NAFLD is often undetected and may progress to non-alcoholic steatohepatitis, liver fibrosis and cirrhosis. Interestingly, the NAFLD prevalence did not differ between the Russian group and the non-Russian group in our study populations, so that it was not related to potential differences in lifestyle between the various ethnic groups in the study populations.

When the observations made in our study are discussed, the limitations of our investigation have to be considered. First, when the findings of investigations on different study populations are compared with each other, differences in the composition of the study populations with respect to the factors associated with the NAFLD prevalence should be considered. Second, the participation rate in the UVOS was relatively low, with 1526 (81.1%) out of 1882 eligible inhabitants participating in the study, and with 1130 (74.0%) of these 1582 individuals having measurements of ALT and AST as basis for the assessment of NAFLD. However, it may be taken into account, that in the old study population of the UVOS with a minimal age of 85 years, the mobility of individuals is markedly decreased, reducing the possibility of individuals participating in studies. In contrast, the participation rate in the UEMS was acceptable with a rate of > 80%. Third, the techniques and methods to diagnose NAFLD differ between studies, in particular between hospital-based studies and population-based investigations^15–17,32,33^. The scientific value of our study would have been improved if we had additionally measured the platelet count, the serum concentration of the gamma-glutamyl-transferase, and the serum albumin concentration, to have additional biomarkers for the presence of NAFLD. It refers in particular for the missing possibility of calculating the FIB (fibrosis)-4 score which is the ratio of the product of age multiplied with the AST concentration (IU/L), divided by the product of the platelet count (10^9^/L) multiplied with the square root of the ALT concentration (IU/L)^32^. It might also have enabled us to determine the fatty liver index as another marker for NAFLD^33^. As another point, the criterion of an AST/ALT ratio of > 1 as criterion for the definition of NAFLD might have selected patients with more advanced fibrosis into the group of individuals with NALD in the current study. The limitations of a population-based study, which as compared to hospital-based investigations have a relatively high proportion of healthy study participants, were the reason for these limitations. Strengths of our project were that the NAFLD prevalence has only scarcely been assessed yet in Eastern Europe and Russia, and that the UVOS is one of the only few studies worldwide including a very old population with an age of 85 + years.

In conclusion, the NAFLD prevalence of 40% in the UEMS and 69.8% in the UVOS corresponds to findings obtained in other world regions and shows the importance of NAFLD, including its associated factors such as age, sex, waist-hip ratio, serum creatinine concentration, prothrombin index, ankle-brachial index, and lower physical activity.

## Data Availability

The datasets generated and/or analyzed during the current study are not publicly available due to ongoing additional statistical analyses, but are available from the corresponding authors on reasonable request.
